# Apixaban Enhances Vasodilatation Mediated by Protease-Activated Receptor 2 in Isolated Rat Arteries

**DOI:** 10.3389/fphar.2017.00480

**Published:** 2017-07-18

**Authors:** Ambra Villari, Giovanni Giurdanella, Claudio Bucolo, Filippo Drago, Salvatore Salomone

**Affiliations:** Pharmacology Section, Department of Biomedical and Biotechnological Sciences, University of Catania Catania, Italy

**Keywords:** apixaban, PAR-2, isolated artery, vasodilatation, endothelium, SLIGRL

## Abstract

Apixaban (APX) is a direct inhibitor of factor X (FXa) approved for prophylaxis and treatment of deep venous thrombosis and atrial fibrillation. Because FXa activates protease-activated receptor 2 (PAR-2) in endothelium and vascular smooth muscle, inhibition of FXa by APX may affect vasomotor function. The effect of APX was assessed *in vitro*, by wire myography, in rat mesenteric resistance arteries (MRAs) and basilar arteries challenged with vasoconstrictors [phenylephrine (PE); 5-hydroxytryptamine (5-HT)], vasodilators [acetylcholine (ACh); sodium nitroprusside (SNP)] or with the PAR-2 peptide agonist SLIGRL. APX (10 μM) reduced the vasoconstriction to PE and 5-HT while did not change the vasodilatation to ACh or SNP. SLIGRL induced concentration-dependent vasodilation in pre-constricted arteries, that was reduced by incubation with the NO inhibitor N^G^-nitro-L-arginine (L-NNA) and abolished by endothelium removal. APX enhanced vasodilation to SLIGRL either in the presence or in the absence of L-NNA, but was ineffective in endothelium-denuded vessels. In preparations from heparin-treated rats (to inhibit FXa) APX did not change the vasodilation to SLIGRL. FXa enzymatic activity, detected in mesentery homogenates from controls, was inhibited by APX, whereas APX-sensitive enzymatic activity was undetectable in homogenates from heparin-treated rats. Immunoblot analysis showed that incubation of MRA or aorta with APX increased the abundance of PAR-2, an effect not seen in MRA from heparin-treated rats or in endothelium-denuded aortas. In conclusion, inhibition of FXa by APX increases vasodilatation mediated by PAR-2. APX may act by inhibiting PAR-2 desensitization induced by endogenous FXa. This effect could be useful in the context of endothelial dysfunction associated to cardiovascular diseases.

## Introduction

New oral anticoagulants (NOACs) have been recently and successfully introduced in clinical practice as an alternative therapy for prophylaxis and treatment of deep venous thrombosis and thrombo-embolic prevention in patients with atrial fibrillation (Pudusseri et al., 2013). At variance with coumarins, such as warfarin, which act as vitamin K antagonist and inhibit post-translational modification of several pro-factors in the coagulation pathway, NOACs directly bind to mature, activated coagulation factors such as thrombin and factor X (FXa). In comparison to coumarins, NOACs present several advantages, such as rapid onset of action, for which they do not need any “bridging” with heparin, their predictable effect which does not require lab monitoring, besides poor interactions with food or drugs, short plasma half-life and improved efficacy/safety ratio (Vanassche et al., 2015). Apixaban (APX) is a direct FXa inhibitor, able to bind and inhibit both free and prothrombinase-bound FXa (Luettgen et al., 2011).

Besides coagulation and its direct action on platelets, both thrombin and FXa are able to elicit a number of responses in vascular endothelium, like shape and permeability changes, stimulation of prostaglandin and cytokine production, vasomotor responses, which may represent an important link between tissue damage and hemostatic and/or inflammatory responses (Coughlin, 2005). These actions are, at least partly, mediated by a subfamily of G protein coupled receptors, ubiquitously distributed, called protease activated receptors (PARs) whose peculiar feature is a tethered (anchored) ligand at the N-terminus in their extracellular domain, unmasked via proteolytic cleavage (Hollenberg and Compton, 2002). There are four known PARs, numbered from 1 to 4. Thrombin can activates all PARs but PAR-2; this latter is activated by trypsin, tryptase, and FXa (Hollenberg and Compton, 2002). PARs can be also activated by relatively short synthetic peptides bearing the sequence of specific endogenous tethered ligands (Vu et al., 1991; Hollenberg and Compton, 2002), also called PAR-activating peptides, which allows the investigations of PAR signaling across tissues. Worthy of note, upon activation PARs undergo internalization and down-regulation, as most G-protein coupled receptors do, but with some distinct features that may depend on the long-lasting stimulation exerted by the tethered ligand (Trejo, 2003).

In recent years, several studies have investigated the actions of FXa beyond coagulation. FXa is able to elicit pathways involved in both physiological and pathophysiological processes, and to act on a wide range of cell types, through the activation of both PAR-1 and PAR-2, including endothelial and vascular smooth muscle cells (Borensztajn et al., 2008) (VSMCs). FXa may affect vascular tone through PAR-1- and PAR-2-mediated signaling (Schaeffer et al., 1997; Bae et al., 2010; Rana et al., 2012). Since APX mechanism of action relies on inhibition of FXa activity, it is likely to induce a change in vascular PAR activation and/or expression on cell surface (Suen et al., 2012); subsequent changes in vasomotor function, if occurring *in vivo*, may have beneficial or detrimental consequences on regional blood flow and/or on systemic blood pressure, depending on whether they preferentially go toward a vasodilatory or vasospastic phenotype. However, no studies have so far investigated potential vasomotor effects of NOACs.

In this study we used isolated rat arteries [mesenteric resistance artery (MRA); basilar artery (BA)] as an intact, native system, to test the hypothesis that inhibition of FXa by APX affects vasomotor function. In particular we assessed the vasomotor responses to vasoconstrictors and vasodilators, elicited in the presence and in the absence of APX. Furthermore, we analyzed the vasomotor responses to PAR-2 stimulation, in the presence and in the absence of APX. The results indicate that inhibition of FXa by APX increases vasodilatation mediated by PAR-2 receptors, an effect that seems specifically related to inhibition of PAR-2 downregulation induced by endogenous FXa.

## Materials and Methods

### Preparation of Vessels and Analysis of Vascular Responses

Animal use was approved by the subcommittee for research and animal care at the University of Catania according to guidelines from Italian Ministry of Health. Male Sprague Dawley rats (Harlan, Udine, Italy; 300–350 g) were killed by CO_2_ asphyxiation. MRA (3rd order) and BA were removed, put in physiological salt solution (PSS; composition, mM: NaCl, 118; KCl, 4.6; NaHCO_3_, 25; MgSO_4_, 1.2; KH_2_PO_4_, 1.2; CaCl_2_, 1.2; glucose, 10; EDTA, 0.025), dissected free of adventitia and/or meninges and cut in segments (2 mm length). In some experiments, in order to remove endothelium, arteries were cannulated and perfused with 200 μl 0.03% Triton X-100, followed by 200 μl PSS at a constant flow of 2 ml/h with a perfusion syringe pump (KD Scientific, 100 Series, Holliston, MA, United States).

Following dissection, each arterial segment was mounted in a wire myograph (610 M, Danish Myo Technology, Aarhus, Denmark), by using 40 μm diameter stainless steel wire, for isometric record of contractile force. After mounting, each preparation was equilibrated unstretched for 30 min, in PSS, maintained at 37°C and aerated with a gas mixture 95% O_2_ – 5% CO_2_. The normalized passive resting force and the corresponding diameter were then determined for each preparation from its own length-pressure curve, as previously described (Salomone et al., 2009; Li Volti et al., 2011). Contractile responses were recorded into a computer, by using a data acquisition and recording software (Myodaq and Myodata, Danish Myo Technology). After normalization and 30-min equilibration in PSS, the preparations were stimulated with isotonic depolarizing KCl rich solution, in which part of NaCl had been replaced by an equimolar amount of KCl (composition in mM: NaCl, 22.6; KCl, 98.8; NaHCO_3_, 25; MgSO_4_, 1.2; KH_2_ PO_4_, 1.2; CaCl_2_, 1.2; glucose, 10; EDTA, 0.025, pH 7.4 at 37°C). After washout and 30 min recovery with or without 10 μM APX, the preparations were challenged with phenylephrine (PE, 10 nM–1 μM) or 5-hydroxytryptamine (5-HT, 1 nM–1 μM); once the contractile response had reached a steady state, cumulative concentrations of acetylcholine (ACh, 1 nM–1 μM in MRA, 1 nM–10 μM in BA) were added to the organ bath to assess endothelium-dependent relaxation. Endothelium independent relaxation was assessed by challenging the preparations with cumulative concentrations of the NO-donor sodium nitroprusside (SNP, 1 nM–1 μM in MRA, 1 nM–10 μM in BA), in the presence of the inhibitor of NO synthase N^G^-nitro-L-arginine (L-NNA, 0.1 mM).

To investigate vessel relaxing responses to PAR-2 receptor stimulation, preparations pre-incubated for 30 min with or without 10 μM APX, were pre-constricted with 1 μM PE or 1 μM 5-HT and, once the contraction had reached the steady state, challenged with cumulative concentration of the peptide agonist SLIGRL (10 nM–10 μM in MRA, 100 nM–100 μM in BA).

### FXa Enzymatic Activity

FXa enzymatic activity was evaluated by using a chromogenic substrate assay, Biophen CS-11 (Hyphen BioMed, Neuville-Sur-Oise, France), according to the manufacturer’s instructions. Briefly, mesentery, including mesenteric artery and its branches, was removed from rats injected with saline (control) or 5,000 U/kg low molecular weight heparin 1 h before sacrifice, and immersed in an ice cold buffer solution (0.05 mM Tris, 300 mM NaCl, pH 7.4). Thereafter, tissues were homogenized in ice with a glass potter and centrifuged at 500 ×*g* for 10 min at 4°C. The lower aqueous phase was recovered and assessed in the chromogenic assay. Reactions were assembled in a final volume of 200 μl, which included 20 μl of lysate (lower aqueous phase), 1 or 10 μM APX, 0.36–2.88 mM of chromogenic substrate, in Tris – NaCl buffer. Blanks with the chromogenic substrate at different concentrations but not including the lysate, were run to evaluate and subtract the background absorbance. Reactions were incubated at 37°C for 1 h and stopped by adding 50 μl 2% citric acid; absorbance was measured at 405 nm in a plate reader (VariosKan, Thermo Fisher Scientific, Waltham, MA, United States). Data are reported after appropriate subtraction of background.

### Immunoblot

Mesenteric resistance arteries (3rd order) were taken from rats injected with saline (control) or 5,000 U/kg low molecular weight heparin 1 h before sacrifice. When testing the endothelium-dependence of the effect of APX, in order to efficiently remove endothelium we used rat aortas instead of MRA; for this purpose aorta was opened longitudinally, pinned in a dissection plate in PSS, and gently scraped with a razor blade, five times up and down. Arteries were incubated in PSS for 30 min, with or without 10 μM APX and subsequently homogenized in RIPA buffer supplemented with protease inhibitor (1:100 dilution; Sigma-Aldrich, St. Louis, MO, United States) as previously described (Salomone et al., 2014). The protein content of the homogenate was quantified by Breadford assay; 40 μg proteins were subjected to SDS-PAGE and blotted as described elsewhere (Giurdanella et al., 2015). Membranes were incubated with primary mouse monoclonal antibody against total PAR-2 (SAM11) at 1:500 dilution (Abcam, Cambridge, United Kingdom). The membranes were then incubated with secondary goat antimouse IRDye 800 conjugated antibody at 1:20,000 dilution, purchased from LI-COR (Lincoln, NE, United States) for 1 h at room temperature. The immune complexes were detected by Odyssey imaging system (LI-COR). All blots were controlled for equal loading by probing with GAPDH rabbit polyclonal antibody (1:1,000 dilution; Cell Signaling Technology, Danvers, MA, United States). Efficient removal of endothelium was confirmed by probing the membrane with a rabbit polyclonal antibody against Von Willebrand factor (Abcam).

### Drugs and Reagents

Phenylephrine, ACh, 5-HT, SNP, L-NNA, were from Sigma-Aldrich. These drugs were dissolved at 10 mM in aqueous stock solutions and further diluted in water or directly in physiological salt solution, as required to reach the final concentration. APX was a gift from Bristol-Myers Squibb (New Brunswick, NJ, United States); it was dissolved at 10 mM in dimethyl sulfoxide (DMSO; Sigma-Aldrich) and then further diluted in aqueous solution to reach the final concentration (1 or 10 μM), containing 0.1% DMSO, according to Wong et al. (2008).

The PAR-2 agonist SLIGRL was synthesized by GeneCust (Dudelange, Luxembourg); it was dissolved at 10 mM stock solution and further diluted in PSS as appropriate.

### Statistical Analysis

Data in concentration-contraction curves are expressed as a percentage of K^+^-induced vasoconstriction against a log molar concentration of drug. Each set of data points was curve-fitted by a non-linear regression, best-fit, sigmoidal dose-response curve with no constraints using GraphPad Prism (GraphPad Software, San Diego, CA, United States). Data in concentration-relaxation curves are expressed as residual tone in percent of the steady-state tone induced by PE or 5-HT, measured before the challenge with the vasodilator. Each curve represents the mean of at least eight individual preparations from a minimum of four rats. For each set of experiments, n is expressed as number of preparations.

Whole curves were compared by two-way analysis of variance (ANOVA). Statistical significance was set at *P* < 0.05. The concentration-response curves to PE, 5-HT, ACh, SNP, SLIGRL, with or without APX, were carried out in parallel, i.e., they represent comparisons between the very same run/challenge.

## Results

### Effect of APX on Agonist-Induced Vasoconstriction

**FIGURE 1:**
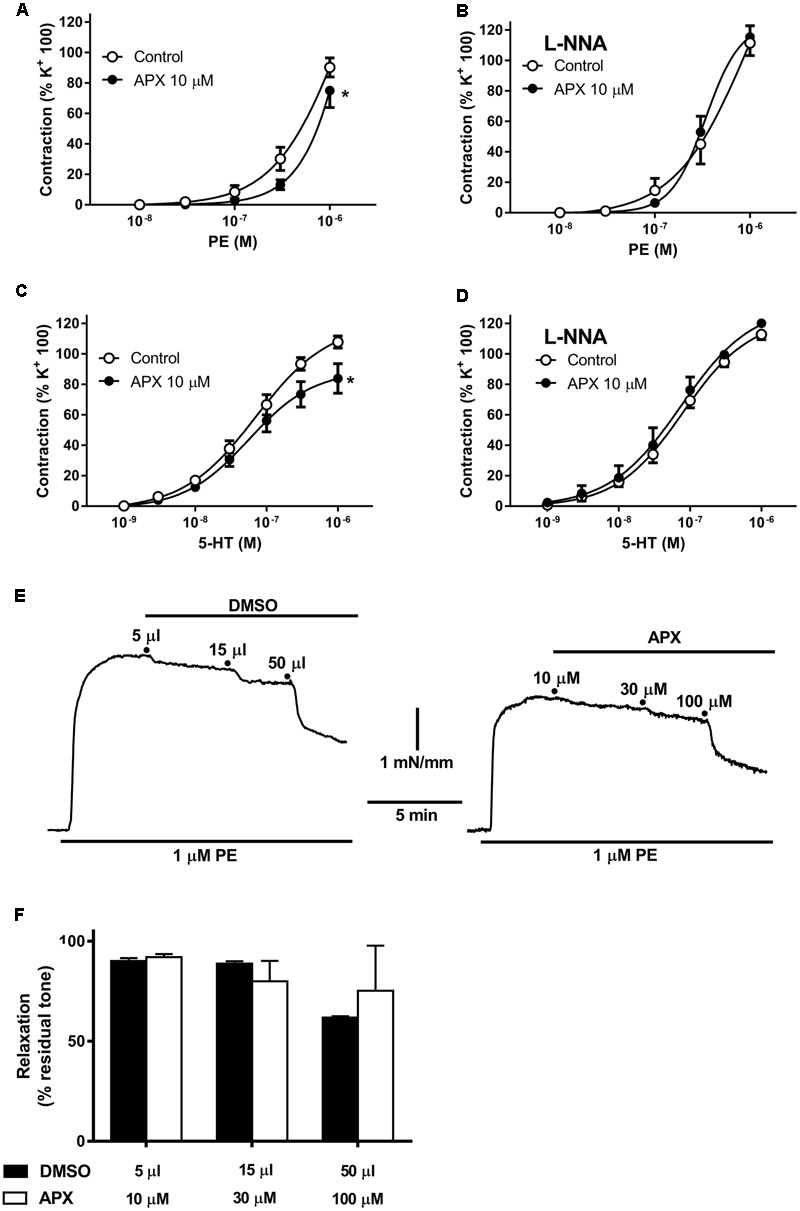
Contractile responses to phenylephrine (PE) and to 5-hydroxytryptamine (5-HT) in isolated mesenteric resistance artery (MRA) **(A,B)** and basilar artery (BA) **(C,D)**; effects of apixaban (APX) and/or N^G^-nitro-L-arginine (L-NNA, 0.1 mM). Notice the reduced vasoconstriction to PE or 5-HT in the presence of APX and in the absence of L-NNA, to block endogenous NO. **(E,F)** Effect of direct addition of APX (10–100 μM) to preparations pre-constricted by 1 μM. PE comparison to the effect of corresponding volumes of vehicle (5–50 μL DMSO). Data are the mean ± SEM of 8–12 independent measurements. ^∗^*P* < 0.01 versus Control. Two-way ANOVA.

Mesenteric resistance artery and BA segments mounted in a wire myograph were first constricted by exposing to a 100 mM KCl-depolarizing solution. Preparations were subsequently incubated with or without APX (10 μM) before being challenged with cumulative concentrations of PE or 5-HT. As shown in **Figures 1A,C**, both PE and **5**-HT elicited concentration-dependent contraction in MRA and BA, respectively, either in control or following incubation with APX; however, APX reduced the vasoconstriction (*P* < 0.05, **Table 1**) to agonists in both arteries. Incubation in the presence of L-NNA (0.1 mM), an inhibitor of NO synthases, augmented the contractile responses to PE and 5-HT (**Figures 1B,D**); furthermore, in the presence of L-NNA APX did not significantly change the contractile responses to vasoconstrictor agonists in either MRA or BA (**Table 1**).

**Table 1:** Pharmacological parameters of vasomotor responses to phenylephrine (PE), 5-hydroxytryptamine (5-HT), acetylcholine (ACh) and sodium nitroprusside (SNP) in isolated mesenteric resistance arteries (MRAs) and basilar artery (BA): effects of apixaban (APX).

	pD_2_	EC_50_ (μM)	E_max_ (% K^+^)	*P*-value
**PE MRA**				
Control APX	5.86 ± 1.48 5.59 ± 7.46	1.40 (0.01–129) 2.59 (very wide)	226 ± 525 Not determined	0.015
**5-HT BA**				
Control APX	7.15 ± 0.10 7.25 ± 0.15	0.07 (0.04–0.11) 0.06 (0.03–0.11)	120 ± 10 89 ± 10	0.001
**PE MRA + L-NNA**				
Control APX	6.01 ± 1.19 6.47 ± 0.08	0.98 (0.04–254) 0.34 (0.24–0.49)	222 ± 351 124 ± 16	0.879
**5-HT BA + L-NNA**				
Control APX	7.10 ± 0.08 7.13 ± 0.14	0.08 (0.05–0.12) 0.07 (0.04–0.15)	123 ± 8 132 ± 15	0.092

	**pD_2_**	**EC_50_ (nM)**	**E_max_ (% tone)**	***P*-value**

**ACh MRA**				
Control APX	7.89 ± 0.09 7.99 ± 0.09	0.01 (0.01–0.02) 0.01 (0.01–0.01)	4.8 ± 4.8 5.2 ± 4.0	0.503
**ACh BA**				
Control APX	6.94 ± 0.32 7.29 ± 0.12	0.12 (0.03–0.51) 0.05 (0.03–0.09)	59.8 ± 7.9 60.4 ± 2.1	0.056
**SNP MRA**				
Control APX	7.74 ± 0.02 7.75 ± 0.05	0.02 (0.02–0.02) 0.02 (0.01–0.02)	1.1 ± 1.1 0.5 ± 3.0	0.787
**SNP BA**				
Control APX	7.43 ± 0.10 7.15 ± 0.05	0.04 (0.02–0.06) 0.07 (0.06–0.10)	1.1 ± 3.6 0.7 ± 2.3	0.042

*Pharmacological parameters are from non linear regression; pD2 is the negative logarithm of the concentration producing 50% of the maximum effect, EC_50_ is the concentration producing 50% of maximum effect (95% confidence limits are given in parenthesis), E_*max*_ is the maximum effect (vasoconstriction to PE or to 5-HT in % of vasoconstriction evoked in the same preparations by 100 mM K^+^; vasodilatation to ACh or SNP as % of residual tone). Some preparations were incubated in the presence of N^*G*^-nitro-L-arginine (L-NNA, 0.1 mM) to inhibit NO biosynthesis. P-values refer to comparisons on the whole curves by two-way ANOVA.*

Addition of APX (10–100 μM) to preparations pre-constricted by 1 μM PE did not induce any significant vasodilatation, as compared to the corresponding volume of vehicle (5–50 μL DMSO, **Figures 1E,F**).

### Effect of APX on Endothelium-Dependent and Endothelium-Independent Vasodilatation

**FIGURE 2:**
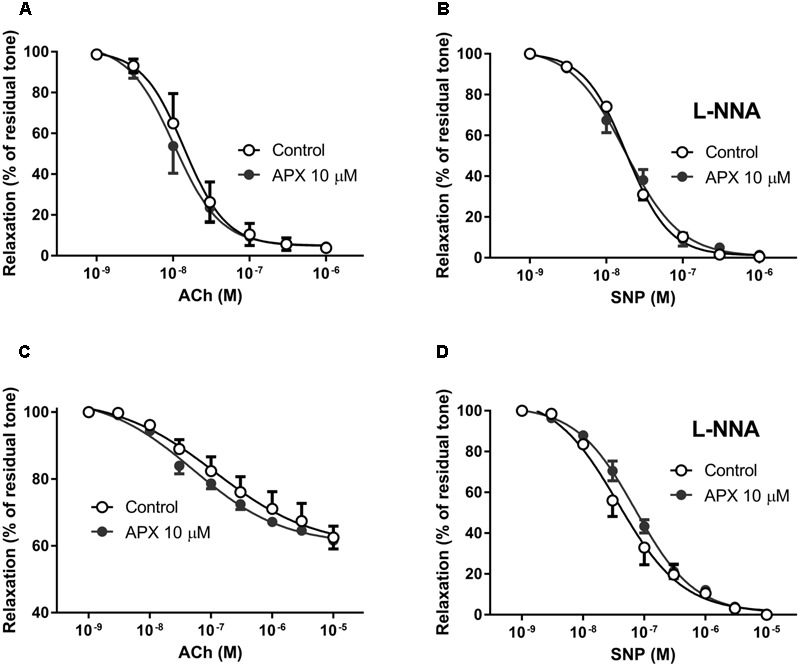
Dilating responses to acetylcholine (ACh) and to sodium nitroprusside (SNP) in isolated MRA **(A,B)** and BA **(C,D)**; effects of apixaban (APX). MRA was pre-constricted with 1 μM phenylephrine, BA was pre-constricted with 1 μM 5-hydroxytryptamine. The vasodilatation to exogenous NO, from the NO-donor SNP was assessed in the presence of N^G^-nitro-L-arginine (L-NNA, 0.1 mM), to block endogenous NO. Data are the mean ± SEM of 8–12 independent measurements.

To examine the potential effect of APX on endothelium-dependent and endothelium-independent vasodilatation, MRA and BA were further challenged with ACh and SNP, added on the top of the steady-state contraction induced by PE or 5-HT, as mentioned above. Notice that, on purpose, we decided to stop the curve to PE at a submaximal concentration, while we run the curve to 5-HT up to its maximum; this protocol was chosen to compromise between the need of analyzing the whole curve of contractile response and the need of analyzing the relaxing responses, these latter being obviously dependent on the intensity of the pre-constriction. Endothelium-dependent vasodilatation to ACh, in fact, was concentration-dependent in both MRA and BA, but was much stronger in MRA that were sub-maximally pre-constricted, achieving almost the basal level tone (i.e., near 100% relaxation, **Figure 2A**), than in maximally pre-constricted BA, where it attained about 40% (**Figure 2C**). Similarly, endothelium-independent vasodilatation to the NO-donor SNP appeared more pronounced in MRA, where relaxations were attained about a half log earlier than in BA, though complete vasodilatation to SNP (i.e., 100% relaxation) was attained in both vessels (**Figures 2B,D**). APX did not significantly modify vasodilatation to ACh or SNP in either MRA or BA (**Table 1**).

### Effect of APX on Vasodilatation Induced by PAR-2 Receptor Stimulation

**FIGURE 3:**
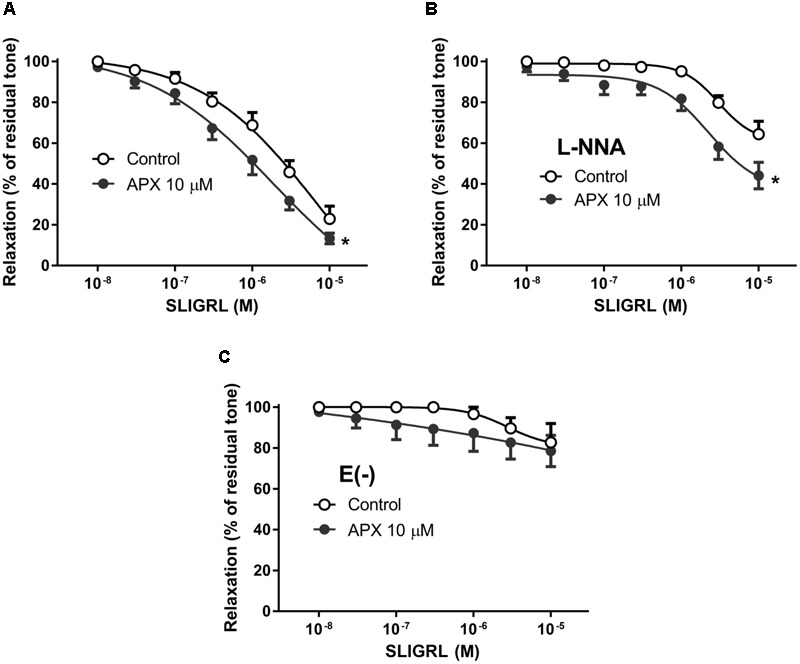
Relaxing responses to the PAR-2 agonist SLIGRL in isolated MRA; effects of apixaban (APX). The vasodilatation to SLIGRL was assessed in intact arteries, in the absence **(A)** or in the presence **(B)** of N^G^-nitro-L-arginine (L-NNA, 0.1 mM), to block endogenous NO, or in endothelium-denuded arteries **(C)**. MRA was pre-constricted with 1 μM phenylephrine. Data are the mean ± SEM of 8–12 independent measurements. ^∗^*P* < 0.01 versus Control. Two-way ANOVA.

**FIGURE 4:**
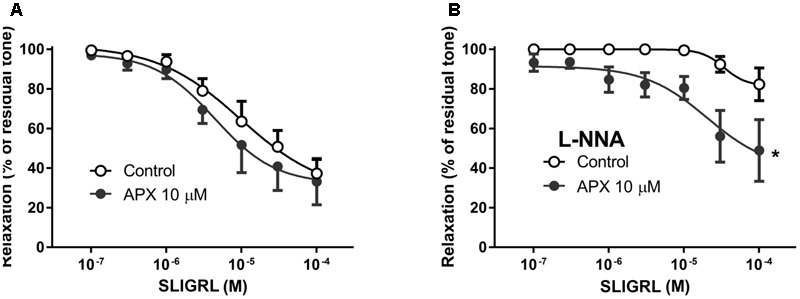
Relaxing responses to the PAR-2 agonist SLIGRL in isolated BA; effects of apixaban (APX). The vasodilatation to SLIGRL was assessed in intact arteries, in the absence **(A)** or in the presence **(B)** of N^G^-nitro-L-arginine (L-NNA, 0.1 mM), to block endogenous NO. BA was pre-constricted with 1 μM 5-hydroxytryptamine. Data are the mean ± SEM of 8–12 independent measurements. ^∗^*P* < 0.01 versus Control. Two-way ANOVA.

The synthetic peptide SLIGRL was used to activate PAR-2 receptors in isolated MRA and BA. PAR-2 receptor is reported to induce mainly vasodilatation (Sobey and Cocks, 1998; McLean et al., 2002); consistently, we did not observe any increase in basal tone following addition of SLIGRL to the PSS in the organ bath (not shown). In MRA pre-constricted by PE (**Figure 3A**) as well as in BA pre-constricted by 5-HT (**Figure 4A**) SLIGRL induced concentration-dependent vasodilation. In both vessels, vasodilatation to SLIGRL was substantially reduced by incubation with L-NNA (**Figures 3B, 4B**), while it was abolished by endothelium removal in MRA (**Figure 3C**); this latter finding indicated that the vasodilatation to SLIGRL was only in part NO-dependent, but totally endothelium-dependent, i.e., entirely attributable to stimulation of endothelial PAR-2. As mentioned above, because relaxing responses are heavily dependent on the intensity of the pre-constriction, we could expect more robust relaxations at lower SLIGRL concentrations in MRA (sub-maximally pre-constricted by 1 μM PE) compared to BA (maximally pre-constricted by 1 μM 5-HT), and this was indeed the case (**Figures 3, 4**).

Pre-incubation of intact arteries with APX enhanced vasodilation to SLIGRL, either in the presence or in the absence of L-NNA, though this effect did not reach statistical significance in BA (MRA, *P* < 0.01, MRA with L-NNA, *P* < 0.01; BA, *P* = 0.108, BA with L-NNA *P* < 0.01; **Table 2**); APX was ineffective in endothelium-denuded MRA, where SLIGRL did not induce significant vasodilatation.

**Table 2:** Pharmacological parameters of vasomotor responses to the PAR-2 agonist SLIGRL in isolated mesenteric resistance arteries (MRAs) and basilar artery (BA): effects of apixaban (APX).

	pD_2_	EC_50_ (μM)	E_max_ (% tone)	*P*-value
**MRA**				
Control APX	5.15 ± 1.13 5.74 ± 0.57	7.02 (0.04–1211) 1.83 (0.14–24.3)	42.9 ± 112 23.4 ± 49.0	<0.001
**MRA + L-NNA**				
Control APX	5.52 ± 0.13 5.64 ± 0.23	3.03 (1.70–5.40) 2.27 (0.81–6.31)	60.8 ± 6.9 36.3 ± 14.2	<0.001
**MRA from heparin-treated**				
Control APX	5.98 ± 0.25 6.15 ± 0.30	1.04 (0.33–3.34) 0.71 (0.17–2.92)	-12.3 ± 23.2 -9.9 ± 27.9	0.384
**MRA from heparin-treated + L-NNA**				
Control APX	4.06 ± 7.40 5.19 ± 2.10	86.4 (very wide) 6.45 (very wide)	-146 ± 1318 -10.6 ± 174	0.747
**BA**				
Control APX	5.02 ± 0.36 5.36 ± 0.25	9.65 (1.84–50.6) 4.41 (1.35–14.4)	27.6 ± 21.7 32.1 ± 11.9	0.108
**BA + L-NNA**				
Control APX	4.47 ± 0.26 4.69 ± 0.65	34.1 (10.0–116) 20.3 (1.00–411)	81.7 ± 7.9 39.4 ± 32.5	<0.001

*Pharmacological parameters are from non linear regression; pD2 is the negative logarithm of the concentration producing 50% of the maximum effect, EC_50_ is the concentration producing 50% of maximum effect (95% confidence limits are given in parenthesis), E_*max*_ is the maximum vasorelaxaton expressed as % of residual tone. Some preparations were incubated in the presence of N^G^-nitro-L-arginine (L-NNA, 0.1 mM) to inhibit NO biosynthesis. P-values refer to comparisons on the whole curves by two-way ANOVA.*

**FIGURE 5:**
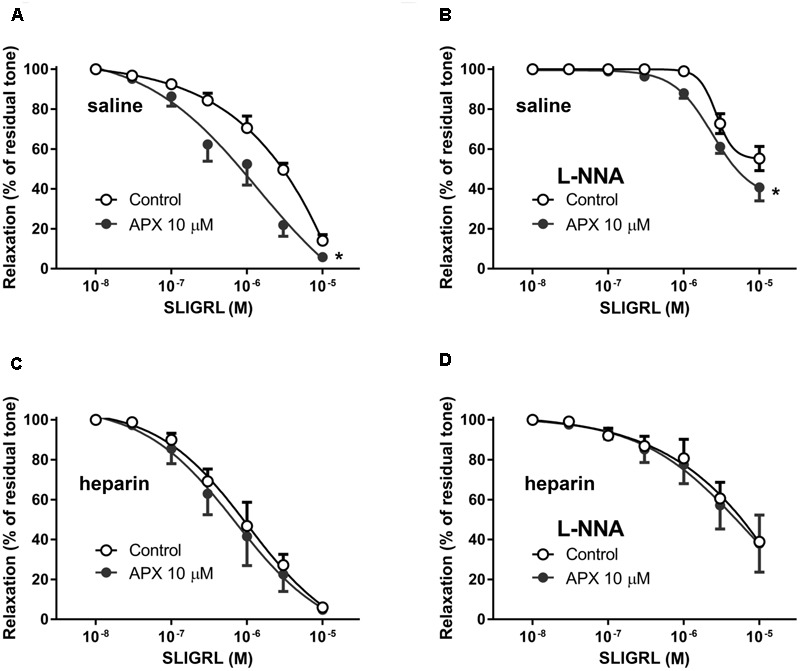
Relaxing responses to the PAR-2 agonist SLIGRL in isolated MRA from Control **(A,B)** and heparin-injected **(C,D)** rats; effects of apixaban (APX). Rats injected with saline (Control) or 5,000 U/kg low molecular weight heparin, 1 h before sacrifice. The vasodilatation to SLIGRL was assessed in the absence or in the presence of N^G^-nitro-L-arginine (L-NNA, 0.1 mM), as indicated, to block endogenous NO. MRA was pre-constricted with 1 μM phenylephrine. Data are the mean ± SEM of 8–12 independent measurements. ^∗^*P* < 0.01 versus Control. Two-way ANOVA.

In order to ascertain whether or not the effect of APX in increasing vasodilatation to SLIGRL was truly attributable to inhibition of FXa, we sought to inhibit FXa *in vivo*, before sacrifice and dissection of arterial preparations, expecting a reduction or a loss of the *in vitro* effect of APX in the absence of FXa activity. Because heparin is well-known to rapidly inhibit FXa (Berry et al., 1998) we compared the vasodilatory responses to SLIGRL of MRA from rats injected *in vivo* with saline (control) or 5,000 U/kg low molecular weight heparin, 1 h before sacrifice. In these preparations we further investigated the effect of *in vitro* treatment with APX. As shown in **Figure 5**, in preparations from animals pretreated with heparin the relaxing effect of SLIGRL was increased compared to saline-treated controls (*P* < 0.01, two-way ANOVA). APX increased the vasodilation to SLIGRL in preparations from controls to the level of those from heparin-pretreated animals, but did not further increase the vasodilation to SLIGRL in preparations from heparin-pretreated animals (**Table 2**).

### FXa Enzymatic Activity and PAR-2 Protein Expression

**FIGURE 6:**
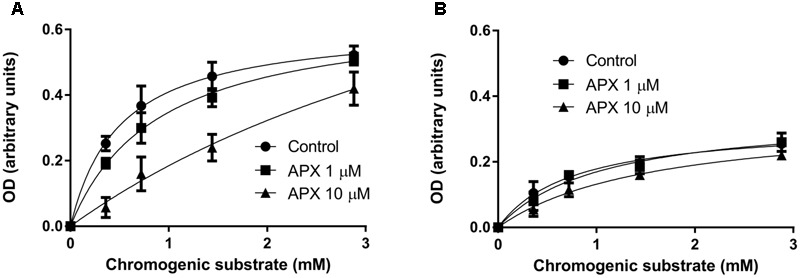
FXa enzymatic activity in homogenates of mesentery from saline-injected rats **(A)** and from heparin-injected rats **(B)**. The mesentery, including mesenteric artery and its branches, was removed from rats injected with saline or 5,000 U/kg low molecular weight heparin 1 h before sacrifice, homogenized and incubated with the chromogenic substrate Biophen CS-11, with or without apixaban (APX, 1 or 10 μM). Notice that in homogenate from control rat apixaban inhibits in an apparent competitive manner the enzymatic activity, whereas in homogenate from heparin-treated rat the enzymatic activity is decreased and barely sensitive to apixaban. Data are reported after appropriate subtraction of background; each concentration point was run in triplicate; the experiment has been repeated twice with similar results.

The change of vasodilatation to PAR-2 stimulation following treatments *in vivo* with heparin or *in vitro* with APX suggested that FXa was available and operating in endothelium, in isolated arterial preparations. To analyze FXa enzymatic activity, homogenates of the whole mesentery, including vessels, were incubated with a chromogenic substrate, specific for FXa (see methods). In preliminary experiments, incubation of increasing concentrations of proteins showed proportional increase of enzymatic activity (not shown). As shown in **Figure 6A**, homogenates from control, saline-injected rats cleaved the chromogenic substrate following a Michaelis–Menten kinetics; APX inhibited the enzymatic activity in an apparent competitive manner. Homogenates from heparin-injected rats cleaved the chromogenic substrate much less efficiently, reaching only about 30–40% of the enzymatic activity estimated in controls (**Figure 6B**). Furthermore, APX did not significantly affect the cleavage of the substrate by these latter homogenates, confirming that the enzymatic activity attributable to FXa (supposedly sensitive to APX) has been effectively removed by *in vivo* heparin pretreatment.

**FIGURE 7:**
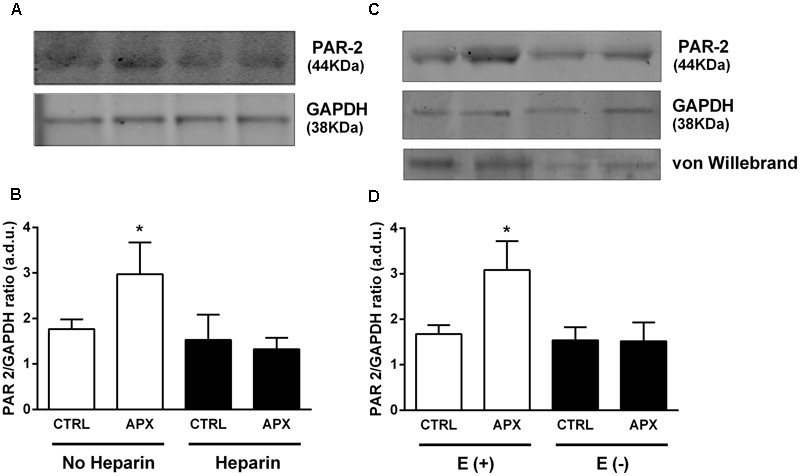
Immunoblot of lysates from **(A,B)** MRAs (from control and heparin-injected rats) and **(C,D)** from aortas (E+, intact and E–, endothelium-denuded); effect of apixaban (APX) on PAR-2 protein level. Arteries were taken from rats injected with saline or 5,000 U/kg low molecular weight heparin 1 h before sacrifice. Arteries were incubated in physiologic salt solution for 30 min, with or without 10 μM APX and subsequently homogenized, subjected to SDS-PAGE, blotted and probed with a mouse monoclonal antibody for PAR-2. The blots were controlled for equal loading by probing with GAPDH rabbit polyclonal antibody; the presence of endothelium was checked by probing with Von Willebrand factor rabbit polyclonal antibody. The experiment was repeated three times; average densitometry values are reported in **(B,D)**. ^∗^*P* < 0.05 versus Control; One-way ANOVA and Tukey’s test.

Based on the known activation of PAR-2 by FXa and on the demonstration of FXa enzymatic activity in mesenteric circulation, to explain the vasomotor effects of APX, particularly on vasodilation induced by PAR-2 stimulation by SLIGRL, we hypothesized that APX, by blocking FXa protected PAR-2 from desensitization and subsequent internalization and degradation. In this respect, we detected PAR-2 by immunoblot analysis in lysates from MRA. MRAs were dissected from either control (saline) or heparin-pretreated rats, incubated in PSS with or without APX, in a condition similar to that used for recording vasomotor responses. Immunoblot analysis showed a single band between 42 and 52 kDa, consistent with a 44 kDa predicted molecular weight of PAR-2. As shown in **Figures 7A,B**, incubation with APX increased the abundance of PAR-2, an effect that was not seen in MRAs from heparin-treated rats. In order to elucidate whether the PAR-2 population sensitive to APX was located in endothelium or in vascular smooth muscle we repeated the PAR-2 immunoblot analysis in intact and endothelium-denuded aortas. Aorta was chosen for technical reason, because endothelium can be efficiently removed by scraping. As shown in **Figures 7C,D**, incubation with APX increased the abundance of PAR-2 in intact aortas; endothelium-denuded aortas showed a sharp reduction of PAR-2 that was insensitive to APX-treatment.

## Discussion

Apixaban is a direct inhibitor of FXa, clinically used to inhibit coagulation and thrombus formation in deep veins or fibrillating atrium (Greig and Garnock-Jones, 2016). However, besides its role in the coagulation cascade, FXa is able to activate PAR-2 receptors, which are expressed in a number of cell types, including endothelial cells and VSMC. Therefore, we hypothesized here that APX would exert vasomotor effects in isolated rat arteries, possibly related to PAR-2 function. This question may have translational relevance because, if an APX-related change in vascular tone is observed in our *in vitro* model, then the likelihood that such an effect occurs *in vivo* in humans must be taken into account, particularly when considering that the duration of APX-treatment spans several weeks. Our results indeed show that APX reduced the vasoconstriction to PE and 5-HT and enhanced the vasodilatation to the PAR-2 agonist SLIGRL, while it did not change the vasodilatation to ACh or SNP, in both MRA and BA. APX is a very selective blocker of FXa, showing a more than 30,000-fold higher selectivity for FXa over other human coagulation proteases (Wong et al., 2011); in rat, however, APX is 10–20 times less active than in human and rabbit, requiring concentrations in the micromolar range to achieve 50% decrease in thrombus weight in various *in vivo* thrombosis models (Schumacher et al., 2010). Based on these published data, we choose the 10 μM APX concentration to maximize the likelihood of observing vasomotor effects related to FXa blockade in isolated rat arteries. First we observed that APX produced a modest, but statistically significant decrease in the vasoconstrictor response to agonists in both MRA and BA; this effect was not present any more when NO production had been blocked by L-NNA, indicating that this effect was dependent on NO production. Our main working hypothesis is that APX, by inhibiting FXa reduces the cleavage of the PAR-2 tethered ligand and the downstream signaling events responsible for PAR-2 down regulation. If this view is correct, in the presence of APX a higher number of endothelial PAR-2 stimulates eNOS to produce NO, which partially inhibit vasoconstrictor responses to agonists in isolated arteries. This hypothesis implies that at least some of PAR-2 receptors present in the endothelium of our isolated preparations are activated by an APX-insensitive mechanism (i.e., a protease different from FXa, given that the presence of APX hinders PAR-2 activation by FXa) and/or are constitutively active. It has been shown that PAR-1 and PAR-2 form heterodimers; within these heterodimers the N-terminus of PAR-1 cleaved by thrombin generates a tethered ligand able to bind and activate PAR-2 *in trans* (Lin and Trejo, 2013). Thus, in our system, APX may increase the availability of PAR-2 in endothelium, by inhibiting their down regulation, while a PAR-1-derived ligand may still be available for *trans* activation of those PAR-2 recruited in heterodimers. Alternatively, other unknown mechanisms, involving different proteases and/or endogenous ligands derived from receptor shedding might conceivably take place. The fact that the vasodilation induced by ACh was not modified by APX indicates that this drug does not affect the mechanism linking muscarinic receptor stimulation to eNOS activation and reinforces the view that the reduction of contractile responses to vasoconstrictor agonists is specifically linked to FXa inhibition and possibly PAR-2 availability on endothelium. On the other hand, endothelium-independent vasodilatation to the NO-donor SNP was also unaffected by APX, indicating that the signaling machinery downstream of NO is not sensitive to APX.

In order to directly test the involvement of PAR-2 into the effect of APX, we further investigate the effect of PAR-2 stimulation by SLIGRL. In both MRA and BA, SLIGRL produced concentration-dependent vasodilatation, an effect partially blocked by L-NNA and abolished by endothelium removal, as previously observed by others (Sobey et al., 1999; Trottier et al., 2002; Kawabata et al., 2004). The vasodilatation to SLIGRL has been also reported to occur *in vivo*, in a cranial window model, an effect again sensitive to NOS inhibitors (Sobey and Cocks, 1998; Sobey et al., 1999). In our experimental setting, in both MRA and BA, the PAR-2-mediated vasodilatation, as observed with SLIGRL, showed a component resistant to NO-blockade, but still endothelium-dependent, because it was abolished by endothelium removal. PAR-2 activation causes endothelium-dependent coronary vasodilation in isolated perfused rat heart, that is abolished after endothelium removal, but not by treatment with L-NAME or indomethacin (McLean et al., 2002). Such an effect of SLIGRL has been related, at least in some vascular districts, to endothelium release of endothelium-derived hyperpolarizing factor (EDHF) (Kawabata et al., 2003). PAR-2 receptor has been localized by immunohistochemistry in endothelium of human coronary arteries (Hamilton et al., 2001); some reports, however, have identified expression of PAR-2 receptors in VSMC in mouse aorta, where they may have functional relevance for vascular tone (Roviezzo et al., 2005). Furthermore, PAR-2 is expressed in non-vascular smooth muscle, such as bronchi, where it induces bronchoconstriction (Schmidlin et al., 2001). The effect of APX on PAR-2-mediated vascular responses *in vitro*, may therefore have a different impact in different vessels/species, according to the localization of PAR-2 in endothelium, VSMC or both; *in vivo*, a further factor to take into account is the access/distribution of circulating APX to the relevant cellular compartment. The action of APX we observed *in vitro*, in isolated arteries, indicates that FXa was present in our system. In general, the assembly and activation of the prothrombinase complex of coagulation, which includes FXa and FVa, is thought to take place mainly at the membrane of activated platelets, i.e., mostly within the thrombus. Recently, however, the prothrombinase complex has been reported to extend into the endothelial surface, largely beyond the thrombus (Ivanciu et al., 2014). Consistently, we detected FXa enzymatic activity in homogenates of the whole mesentery which was inhibited by APX in an apparent competitive manner; as expected for FXa, such an activity was substantially reduced in homogenates from heparin-injected rats. FXa recruitment on endothelial surface might be induced by noxious stimuli, such as inflammatory cytokines (Ivanciu et al., 2014), but it has also been reported to occur spontaneously, in a time-dependent manner, at least *in vitro*, in normal human umbilical vein endothelial cells (Sugo et al., 1995); moreover, FXa has been demonstrated to be present in the surface of cultured endothelial cells (Stavik et al., 2016). It is quite possible that some activation of endothelial cells in MRA and BA occurred, in our system, during the sacrifice of the animal and the dissection of the arteries; however, the scope of this work was not to analyze the extent of the activation and recruitment of FXa into the endothelium, nor the mechanism responsible for such activation if any, but rather to assess whether FXa blockade by APX impacts on vasomotor responses and the underlying mechanism. The present data indeed indicate that FXa blockade by APX enhances PAR-2 mediated responses, presumably due to decreased PAR-2 desensitization. PAR-2 desensitization follows, in some respect the general mechanisms of G-protein coupled receptor desensitization, involving phosphorylation and β-arrestin binding followed by internalization in a clathrin-coated vesicle, lysosomal degradation or recycling to the plasma membrane (Hollenberg and Compton, 2002; Jung et al., 2016; Schoneberg et al., 2016). An important difference with most receptors activated by soluble ligands is that here the ligand, being tethered and not free of diffusing away, exposes the receptor to a persistent stimulation, supposedly more efficient in inducing desensitization (Schoneberg et al., 2016). Such a prediction is in fact confirmed by experimental evidence of transient PAR-2 stimulation followed by rapid desensitization (Jung et al., 2016). As mentioned above, our hypothesis was that PAR-2 desensitization induced by FXa was inhibited by APX. To assess the availability of PAR-2 in MRA we carried out immunoblot analysis following incubation with APX. This experiment revealed an increased abundance of PAR-2 following incubation with APX, consistent with the idea that APX inhibited PAR-2 stimulation and subsequent down regulation. Interestingly, when the same experiment was carried out in MRA from heparin-treated rats the effect of *in vitro* incubation with APX on PAR-2 abundance was lost. This latter finding suggests that in isolated MRA a protease responsible for PAR-2 activation and desensitization, presumably FXa, is present and active, whereas such a protease activity is not available any more in MRA from heparin-treated rats. This assumption is consistent with the FXa-related enzymatic activity, detected in homogenates from control rats, undetectable in homogenates from heparin-injected rats, and indicates that the increased vasodilatation to SLIGRL in the presence of APX is attributable again to decreased PAR-2 desensitization by FXa, which, in fact did not occur in MRA from heparin-treated rats. Immunoblot analysis of lysates from endothelium denuded arteries showed a substantial reduction of PAR-2 and a loss of APX effect; this data though obtained in aorta, is consistent with functional experiments in MRA, showing an endothelium dependence of vasodilatation induced by the PAR-2 agonist SLIGRL.

## Conclusion

Inhibition of FXa by APX increases vasodilatation mediated by PAR-2 receptors in isolated rat arteries. APX may act by inhibiting PAR-2 desensitization induced by endogenous FXa. Such an effect, if occurring *in vivo*, may have an impact on blood flow, which would be beneficial in the context of endothelial dysfunction, such as that associated to atherosclerosis, hypertension, coronary disease, ischemic stroke.

## Author Contributions

FD and SS designed the study and wrote the manuscript; AV, GG, CB, SS, implemented the experimental protocols, carried out the experiments and analyzed the data.

## Conflict of Interest Statement

The authors declare that the research was conducted in the absence of any commercial or financial relationships that could be construed as a potential conflict of interest.
